# Effect of Propionate on *Citrobacter rodentium* Infection in Mice by Regulating NleH Expression

**DOI:** 10.1111/jcmm.70216

**Published:** 2024-11-24

**Authors:** Yingying Li, Wenjie Mei, Qinhan Zhang, Li Li, Qiaorong Ji

**Affiliations:** ^1^ Department of Pathophysiology, School of Basic Medical Sciences Xuzhou Medical University Xuzhou Jiangsu China; ^2^ Tengzhou Central People's Hospital Tengzhou Shandong China; ^3^ Xuzhou Medical University Xuzhou Jiangsu China; ^4^ Laboratory of Clinical and Experimental Pathology, School of Basic Medical Sciences Xuzhou Medical University Xuzhou Jiangsu China

**Keywords:** *Citrobacter rodentium*, NleH, propionate

## Abstract

Propionate is one of the main short chain fatty acids in the gut. Previously, we found that propionate significantly down‐regulated the expression of NleH. NleH is a virulence effector secreted by *Citrobacter rodentium* ( *C. rodentium* , *C.r.*) and is essential for its intestinal colonisation and infection. Therefore, this study intends to explore the effect and mechanism of propionate on *C.r.* infection by regulating the expression of NleH. Wild‐type *C.r.* and its NleH mutant (*C.r.* ^*△NleH*^), *E.coli* and its NleH1 mutant (*E.coli* ^*△NleH1*^) were co‐cultured with propionate separately, changes in strain growth and invasion adhesion were detected. Meanwhile, C57BL/6J mice were infected with *C.r.* and *C.r.* ^*△NleH*^ to establish animal model, and propionate intervention was given. Through detecting the invasive and infectious ability of strains in mice and the changes related to colon inflammation, to analyse the effect of propionate on *C.r.* infection by regulating NleH expression. The results showed that propionate can reduce the adhesion of *C.r*. and intestinal damage by down‐regulating NleH expression, meanwhile changes of microbial functional metabolism enhance the resistance to *C.r.* infection in mice.

## 1 Introduction

The global challenge posed by *Escherichia coli* ( *E. coli* ) infection remains significant. In 2019 alone, pathogenic *E. coli* strains were responsible for approximately 800,000 deaths worldwide, ranking first among all pathogens [1]. Currently, the standard approach to treating infection involves the use of antibiotics. However, this treatment is often accompanied by serious issues such as antibiotic abuse and the increasing development of bacterial resistance. Epidemiological statistics indicate that, in recent years in China, the number of deaths due to antibiotic resistance in *E. coli* infection accounts for approximately one‐third of all deaths attributed to *E. coli* infections [2]. Therefore, there is an urgent need for safer alternatives in the treatment of *E. coli* infection.

*Citrobacter rodentium* ( *C. rodentium* , *C.r*.), a mouse‐specific intestinal pathogen, is currently recognised as an established model for studying infections caused by human *Enteropathogenic Escherichia coli* (EPEC) and human *Enterohemorrhagic Escherichia coli* (EHEC) [3]. In our previous research, we observed that propionate may be involved in alleviating *C. rodentium* infection in mice, while the mechanism is unclear [4]. Propionate is one of the primary short‐chain fatty acids (SCFAs) found in the intestine. SCFAs are primarily produced by intestinal microbiota through fermentation of resistant starch and fibre, with acetate and butyrate being the other major SCFAs [5]. Numerous studies have demonstrated the significant role of SCFAs in maintaining intestinal homeostasis [6]. They serve as an essential energy source for intestinal epithelial cells and can inhibit the proliferation of pathogenic bacteria, enhance intestinal barrier function, exert anti‐inflammatory effects [6]. SCFAs have also been shown to play a positive role in colitis induced by DSS and TNBS [7, 8].

Given the above considerations, we previously investigated the impact of propionate on *C. rodentium* infection in mice. It is interesting that we found that propionate can significantly down‐regulate the expression of NleH. NleH is one of the virulence effector factors secreted by *C. rodentium* , and it is a protein that *C. rodentium* inject into the host cell through their own type III secretion system (T3SS) [9]. The function of NleH is related to the colonisation of *C. rodentium* in the intestine. It can induce the disappearance of brush border microvilli, which is the Attaching‐and‐Effacing (A/E) lesions. After the occurrence of A/E lesions, the tight junction structure and permeability between host intestinal cells changed, which lead to the damage of intestinal mucosal barrier, creating conditions for further invasion of bacteria in the intestine [10].

Studies have confirmed that specific NleH knockout can significantly reduce the colonisation of *C. rodentium* in C57BL/6J mice, and supplemented with NleH1 can restore the colonisation of *C. rodentium* to the level of wild‐type [11]. NleH belongs to the T3SS effectors family found in various intestinal pathogens. EPEC O127: H6 E2348/69 and EHEC O157: H7 EDL933 contain two NleH paralogues (NleH1, NleH2). *C. rodentium* contains only one NleH gene, which shares 83% and 81% amino acid sequence identity with EHEC O157: H7 NleH1 and NleH2, respectively. *E. coli* encodes NleH1 protein, which functions equivalently to *C. rodentium* NleH [12, 13]. NleH1 is closely related to intestinal cell signalling pathway. Studies have shown that NleH1 can reduce the activity of NF‐κB induced by IKK‐β, which is related to inhibiting intestinal epithelial cell inflammation in order to promote bacteria itself colonisation [14]. NleH1 can also inhibit the activation of ERK1/2 and p38 in vitro. These proteins are closely related to cell inflammation and apoptosis, and inhibiting the expression of these pathway proteins may be a strategy for bacteria to escape immune defence [15].

The interaction between bacteria and intestinal epithelial cells is an important prerequisite for its pathogenesis. Based on this, we propose a scientific hypothesis that propionate alleviate *C. rodentium* infection may be related to its function of down‐regulating the expression of NleH. Therefore, we apply the *C. rodentium* and its NleH mutant strains to explore the role and mechanism of propionate on *C. rodentium* infection. The relevant results are helpful to elucidate the effect of propionate on intestinal *E. coli* infection, which have great significance for developing new antibacterial drugs.

## 2 Materials and Methods

### 2.1 Reagents

Propionate (C_3_H_5_NaO_2_, HPLC≥ 99%, MW = 96.06, CAS NO. 137–40‐6) was purchased from Shanghai Macklin Biochemical Co. Ltd. (Shanghai, China). *C. rodentium* (strain DBS100, ATCC 51459) and EHEC O157:H7 was obtained from Beijing Beina Chuanglian Biotechnology Co. Ltd. (Beijing, China).

### 2.2 Animals

All procedures involved in the care and use of animals were in accordance with ARRIVE (Animal Research: Reporting of In Vivo Experiments) guidelines and China Practice for the Care and Use of Laboratory Animals. The protocols for animal studies were approved by the Institutional Animal Care and Use Committee of Xuzhou Medical University (IACUC Issue No.202311 T016). All steps were taken to minimise pain and suffering in the animals during the experiments.

Six to eight weeks old female C57BL/6J mice were purchased from Xuzhou Medical University. The protocols for animal studies were approved by the Institutional Animal Care and Use Committee of Xuzhou Medical University. Mice were fed autoclaved food and water, and housed under specific pathogen free (SPF) conditions (Experimental Animal Center of Xuzhou Medical University, Xuzhou, China). Indoor lighting control changes at 12/12 h circadian rhythm, and maintain a constant humidity (45%–65%) and temperature (20°C–22°C).

### 2.3 Strains Culture and Determination of Growth Curve

Wild‐type *C.r*. and its NleH mutant (*C.r*.^*△NleH*^), *E.coli* and its NleH1 mutant (*E.coli* ^*△NleH1*^), a single colony was inoculated with LB medium and the culture conditions are 37°C, 220 rpm, shaking, overnight for 12 h. Each strain counts 5 × 10^8^ CFU bacteria and were inoculated in 96‐well with LB medium with propionate (8 mmol/L) at pH 7.0. The concentration of each well was measured by microplate reader at a wavelength of 600 nm every hour.

Detection of expression level of virulence factor NleH1: *E. coli* (5 × 10^8^ CFU) was inoculated into 2‐3 mL LB liquid medium with different concentrations of propionate (8, 25 mmol/L). And then the total RNA was extracted after overnight culture at 37°C and 220 rpm.

### 2.4 *C.r.*, *C.r.* ^*△NleH*^ Infection and Propionate Treatment

Intestinal infection was induced by oral gavage of *C.r*. and *C.r*.^*△NleH*^ (5 × 10^8^ CFU/0.2 mL) [16]. Mice were randomly divided into six groups, include control group (Cont), propionate group (Pro), *C.r*. infection group (*C.r*.), propionate combined with *C.r*. infection group (*C.r*. + Pro), *C.r*.^*△NleH*^ infection group (*C.r*.^*△NleH*^) and propionate combined with *C.r*.^*△NleH*^ infection group (*C.r*.^*△NleH*^ + Pro). Propionate (150 mmol/L) was added to the drinking water of the propionate intervention groups [8].

### 2.5 Body Weight and Faecal Bacterial Output Measurement

Body weight changes of the mice in each group were monitored every other day from the first day of the *C. rodentium* infection, which are represented as a percentage of the initial weight of each individual mouse. At the same time, the faeces of mice in the infection groups were collected and cultured overnight in selective MacConkey Agar (M8560; Solarbio). Then the colonies were counted after overnight culture at 37°C.

### 2.6 Invasion of Bacteria in Mice

Spleen, liver, small intestine and cecum of infected mice were collected on the 5th and 10th day of infection, then weighed and ground with PBS, and the concentration was adjusted to 0.1 mg/μL. After dilution, 10 μL was taken to MacConkey medium at 37°C for overnight culture, and the bacterial output (CFU/g) was counted.

### 2.7 Histopathology

Mice were euthanized after infection 2 weeks. Colon tissues were collected, measured in length, fixed with 4% paraformaldehyde, embedded in sections, and stained with haematoxylin and eosin (HE) [4]. Besides, the spleen, mesenteric lymph nodes and colon tissue of mice were frozen at −80°C.

### 2.8 Quantitative Real‐Time PCR (qRT‐PCR) Analysis

Total RNA was isolated from frozen colon tissue using traditional methods. cDNA was synthesised according to the instructions of the PrimeScript RT Reagent Kit (G3330‐50, Servicebio). Results were normalised to GAPDH expression and relative quantification was calculated using the 2^−ΔΔ*C*^_*t*_ method. The sequences for the sense and antisense primers that were used for mRNA quantification are listed in Table 1.

**TABLE 1:** qPCR primers sequences.

Species	Genes	Sequences(5′‐3′)
Mouse	ZO‐1	F: GACCAATAGCTGATGTTGCCAGAG
		R: TATGAAGGCGAATGATGCCAGA
	occludin	F: GGCAAGCGATCATACCCAGAG
		R: AGGCTGCCTGAAGTCATCCAC

### 2.9 Determination of Bacterial Adhesion

Cells were cultured in 24‐well plates. When the cells covered the bottom of the well, *C.r*., *C.r*.^*△NleH*^, *E. coli* , *E. coli* ^*△NleH1*^ (MOI = 1000) were cultured with the cells for 6 h. After 6 h, the cells were washed 5 times with PBS and lysed with 500 μL of cold 0.1%TritonX‐100. After mixing, 10 μL of the solution was cultured overnight in LB solid medium and MacConkey medium at 37°C, and the colonies were counted.

### 2.10 16S rRNA Gene Amplification and Sequencing

The faeces of mice in each group were collected before and after infection, the changes in the composition and diversity of intestinal microbiota detected by 16SrRNA sequencing, and the functional and metabolic of microbiota were compared and analysed.

### 2.11 Statistical Analysis

All data are presented as Mean ± SEM and analysed using Prism8 (Graphpad). Mean value comparisons were performed with unpaired student's *t*‐test, one‐way ANOVA with Tukey's multiple comparison test and two‐way ANOVA with Tukey's multiple comparison test according to the structure of data. A value of *p* < 0.05 was considered significant.

## 3 Results

### 3.1 Propionate Improves the Survival Condition of *C.r.* Infected Mice by Down‐Regulating NleH

**FIGURE 1:**
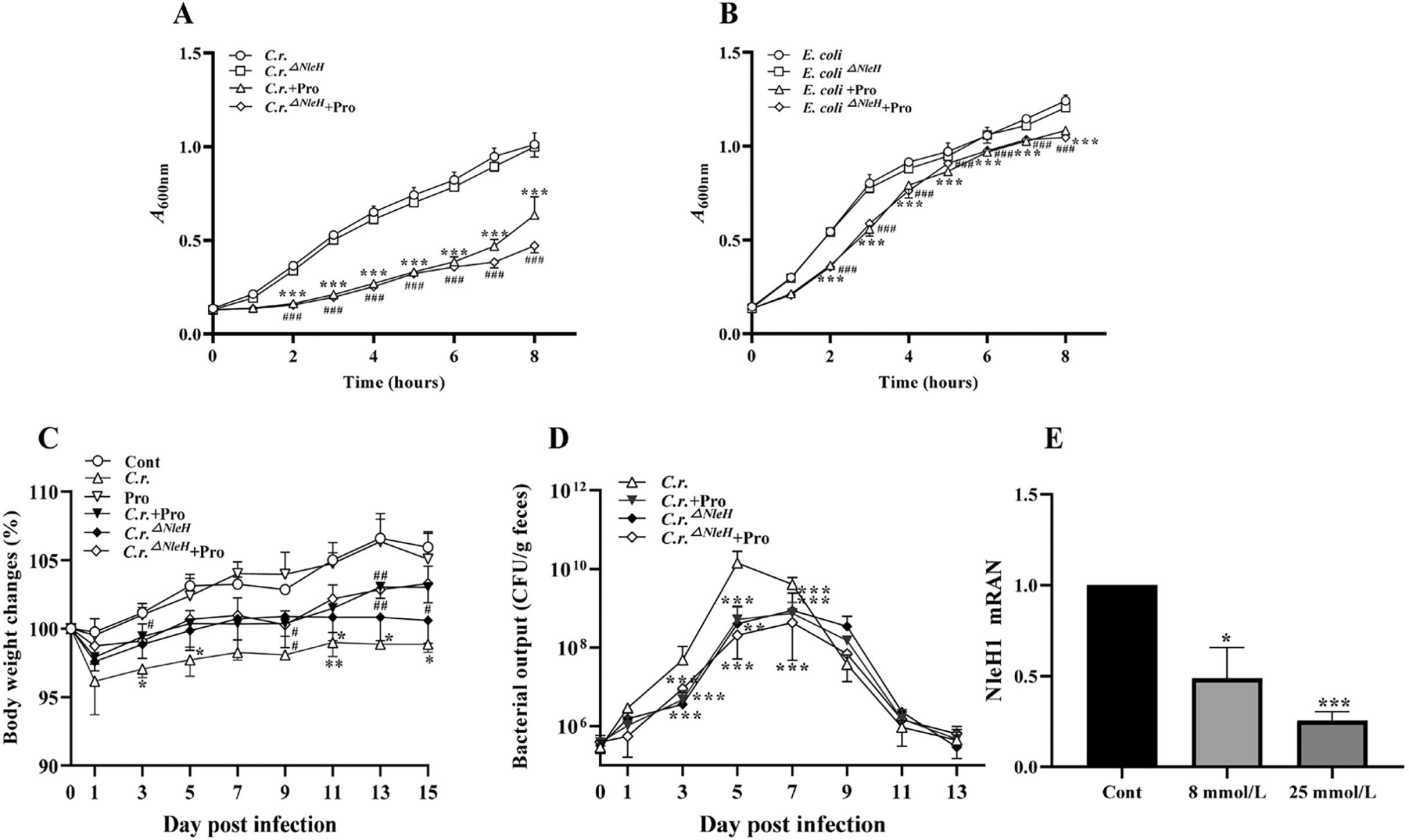
Propionate enhances the physical condition of mice by down‐regulating NleH. (A, B) Propionate (8 mmol/L) was co‐cultured with the bacterial strains, and bacterial growth was monitored every hour. *n =* 3–4, ***p < 0.001 versus *C.r*., *E. coli* ; ### *p* < 0.001 versus *C.r*.^*△NleH*^, *E. coli* ^*△NleH1*^. (C) Applied *C.r*. and *C.r*.^*△NleH*^ to mice, observed the weight changes and bacterial output of mice. *n* = 4–6, *p < 0.05, **p < 0.01, ***p < 0.001 versus Cont; # *p <* 0.05, ## *p <* 0.01 versus *C.r*. (D) The bacterial output of mice. *n* = 4–6, **p < 0.01, ***p < 0.001 versus *C.r*. (E) The expression level of NleH1 in *E. coli* . *p < 0.05, ***p < 0.001 versus Cont.

We cultured the NleH mutant (*C.r*.^*△NleH*^) and NleH1 mutant (*E.coli* ^*△NleH1*^) strains with 8 mmol/L propionate in vitro. First, we detected the growth state of these strains. The results showed that the knockout of NleH or NleH1 did not affect the growth of bacteria, and propionate inhibited the growth of *C.r*., *C.r*.^*△NleH*^, *E. coli* and *E. coli* ^*△NleH1*^ (Figure 1A,B). In addition, we also found that propionate reduced the expression of virulence factor NleH1 in *E. coli* (Figure 1E), which was consistent with our findings that propionate can significantly reduce the expression of NleH in *C.r*. This provides a basis for our follow‐up in vivo and in vitro experiments.

We infected mice with *C.r*. and *C.r*.^*△NleH*^ to prepare the infection model and observe the survival state of mice. The results showed that mice lost weight significantly in *C.r*. group, while the degree of weight loss were reduced in *C.r. +* Pro and *C.r*.^*△NleH*^ group. There was no significant difference between *C.r*.^*△NleH*^ + Pro, *C.r*. + Pro and *C.r*.^*△NleH*^ group. Similarly, compared with *C.r*. group, the intestinal bacterial excretion in *C.r*. + Pro, *C.r*.^*△NleH*^ and *C.r*.^*△NleH*^ + Pro groups were reduced, which indicated that propionate could regulate the expression of NleH and reduce the weight loss and intestinal bacterial excretion in *C.r*. infection (Figure 1C,D).

### 3.2 Propionate Alleviates Colon Inflammation Caused by *C.r.* Infection in Mice by Down‐Regulating NleH

**FIGURE 2:**
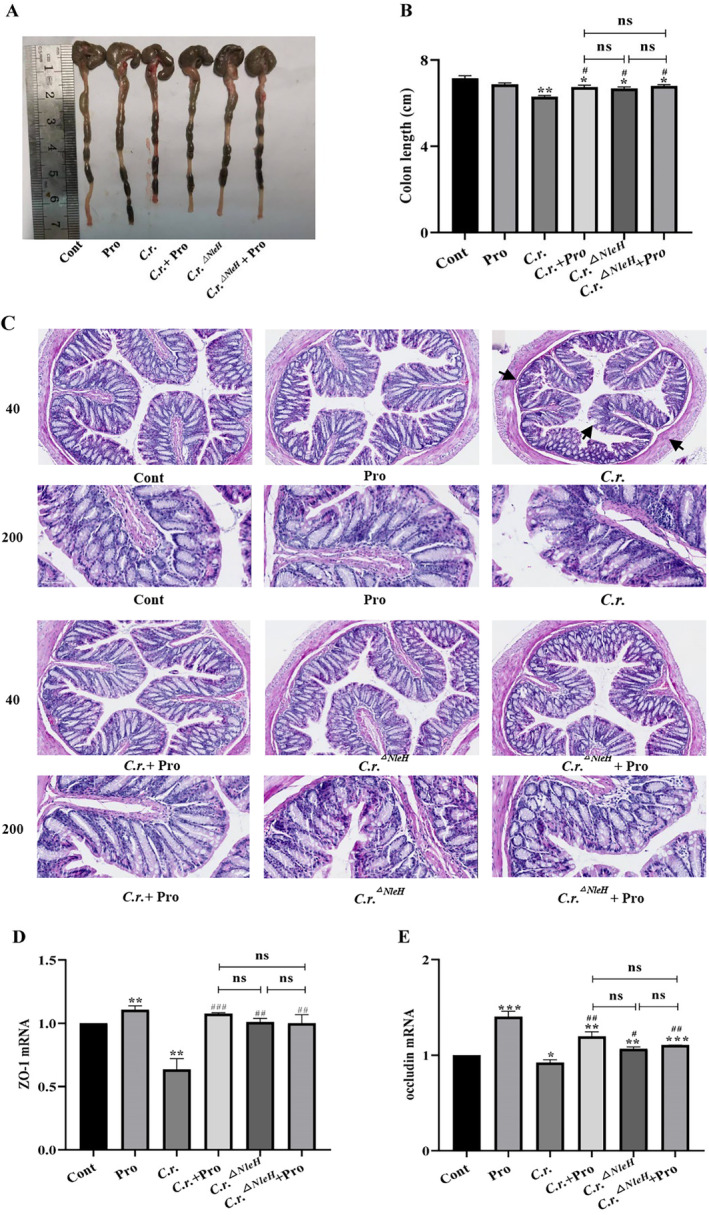
Propionate alleviates colon inflammation by regulating NleH expression. (A, B) Colon length changes of mice. (C) HE pathological staining of colon (40x, 200x). (D, E) The expression level of ZO‐1 and occludin in colon. *n* = 4–6, *p < 0.05, **p < 0.01, ***p < 0.001 versus Cont; # *p* < 0.05, ## *p* < 0.01, ### *p* < 0.001 versus *C.r*.

We detected the length and histopathological changes of the distal colon in mice. The results demonstrated that the colon shortening was most obvious in *C.r*. group, while mice in the *C.r*. + Pro, *C.r*.^*△NleH*^, and *C.r*.^*△NleH*^ + Pro groups all exhibited a low degree of colon shortening, which did not reach statistical significance (Figure 2A,B). Pathological examination revealed severe colon mucosal damage and significant thicken in *C.r*. group, while mice in *C.r*. + Pro, *C.r*.^*△NleH*^ and *C.r*.^*△NleH*^ + Pro groups displayed less thickening of the colon and milder inflammatory damage (Figure 2C). This suggested that propionate can alleviate colon inflammation by regulating the expression of NleH.

We then explored the influence of propionate on the barrier function of the intestinal epithelium. The expression of tight‐junction‐associated proteins in the colon of mice, including ZO‐1and occludin were detected. The results indicated that the expression of ZO‐1 and occludin in colon were decreased of *C.r*. group, while the expression of ZO‐1 and occludin significantly increased in *C.r*. + Pro, *C.r*.^*△NleH*^, and *C.r*.^*△NleH*^ + Pro groups. Also, there was no significantly difference between *C.r*. + Pro, *C.r*.^*△NleH*^, and *C.r*.^*△NleH*^ + Pro groups (Figure 2D,E). This suggested that propionate enhanced intestinal barrier function by regulating the expression of NleH.

### 3.3 Propionate Weakens Bacterial Adhesion by Down‐Regulating NleH

**FIGURE 3:**
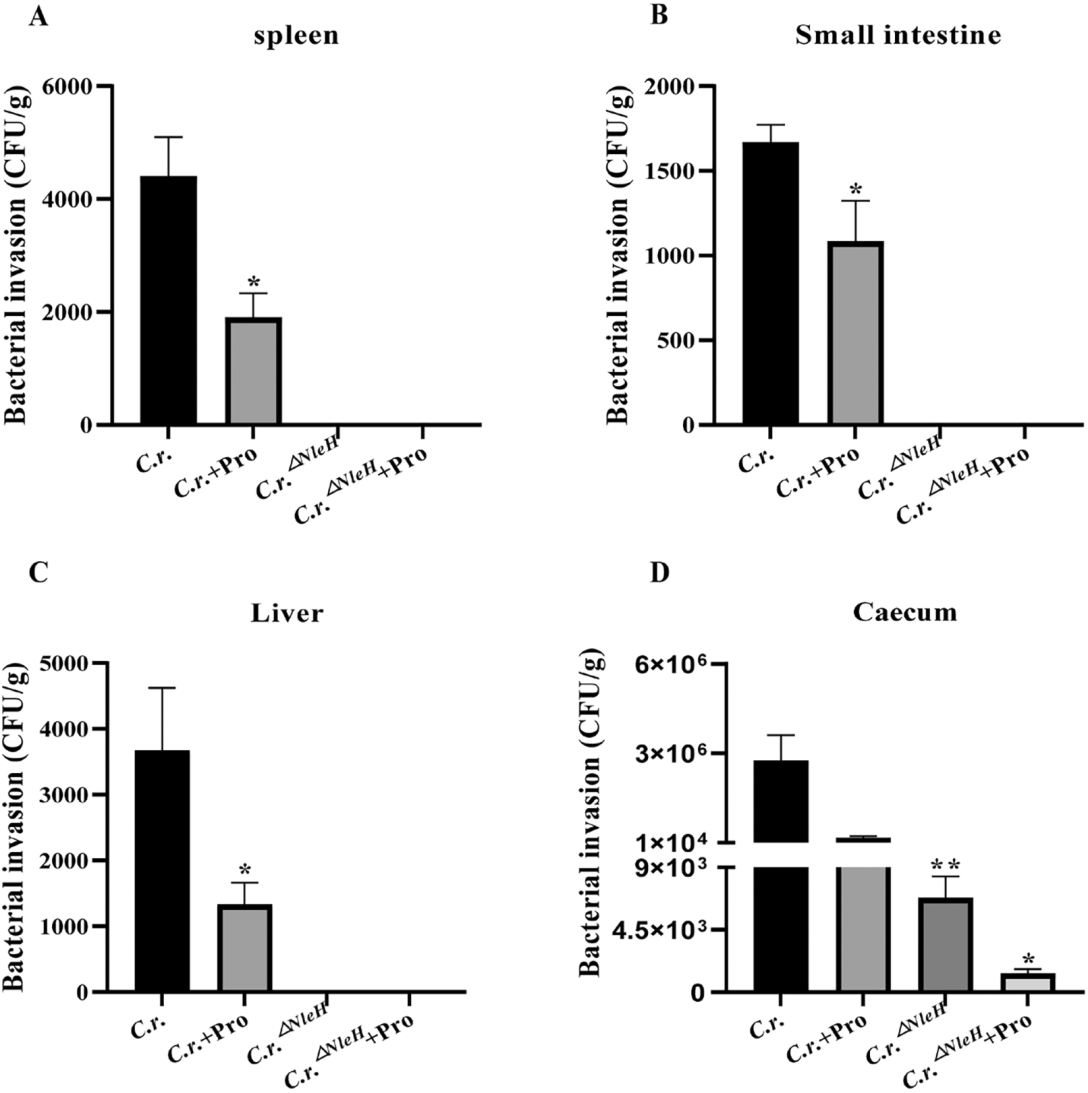
Propionate weakens bacterial adhesion by down‐regulating NleH. The number of bacteria in the spleen (A), small intestine (B), liver (C) and cecum (D) of mice on the 10th day after infection. *n* = 4–6, *p < 0.05, **p < 0.01 versus *C.r*.

**FIGURE 4:**
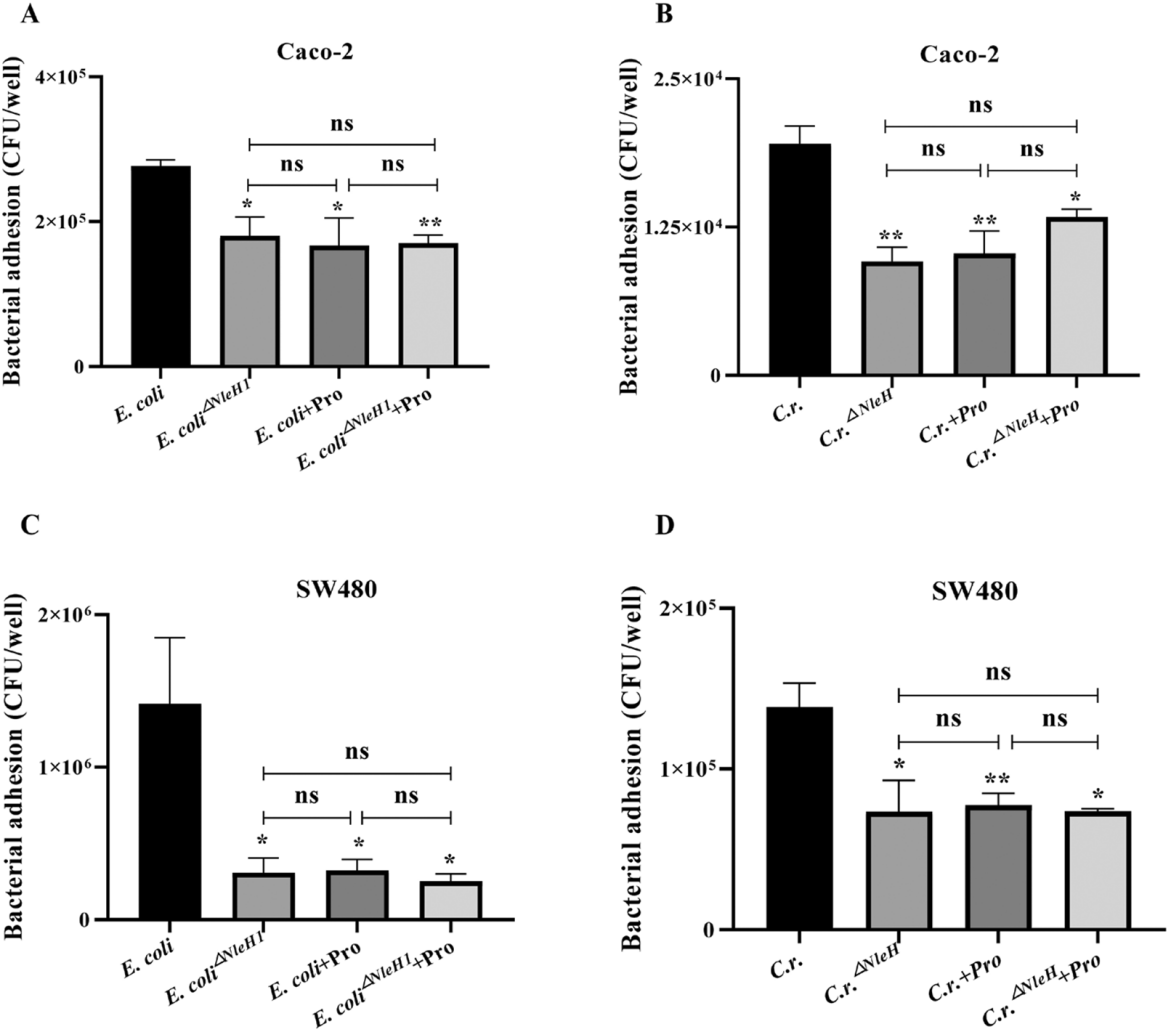
Propionate weakens bacterial adhesion by down‐regulating NleH. *C.r*.^*△NleH*^, *E.coli* ^*△NleH1*^ were co‐cultured with propionate (8 mmol/L) for 6 h, then infected with Caco‐2 (A, B) or SW480 cells (C, D) to detect the adhesion of the strains. *n* = 3–4, *p < 0.05, **p < 0.01 versus *E. coli* , *C.r*.

To investigate the impact of propionate on the invasive capacity of *C.r*., we measured the bacterial count in spleen, liver, small intestine, and caecum of mice on the 5th and 10th day after *C.r*. and *C.r*.^*△NleH*^ infection (Figure 3). However, no invasion of strains was detected on the 5th day after infection, so the results was not shown in this article. Results showed that the absence of NleH also contribute to the reduction of bacterial invasion, and propionate possesses the capacity to attenuate *C.r*. invasion in vivo. Bacterial adhesion testing showed that, the adhesion ability of *C.r*.^*△NleH*^ and *E. coli* ^*△NleH1*^ to Caco‐2 and SW480 cells is reduced, also propionate treatment did not improve this change (Figure 4).

### 3.4 Propionate Regulates the Metabolic Functions of Microbiota by Down‐Regulating NleH

**FIGURE 5:**
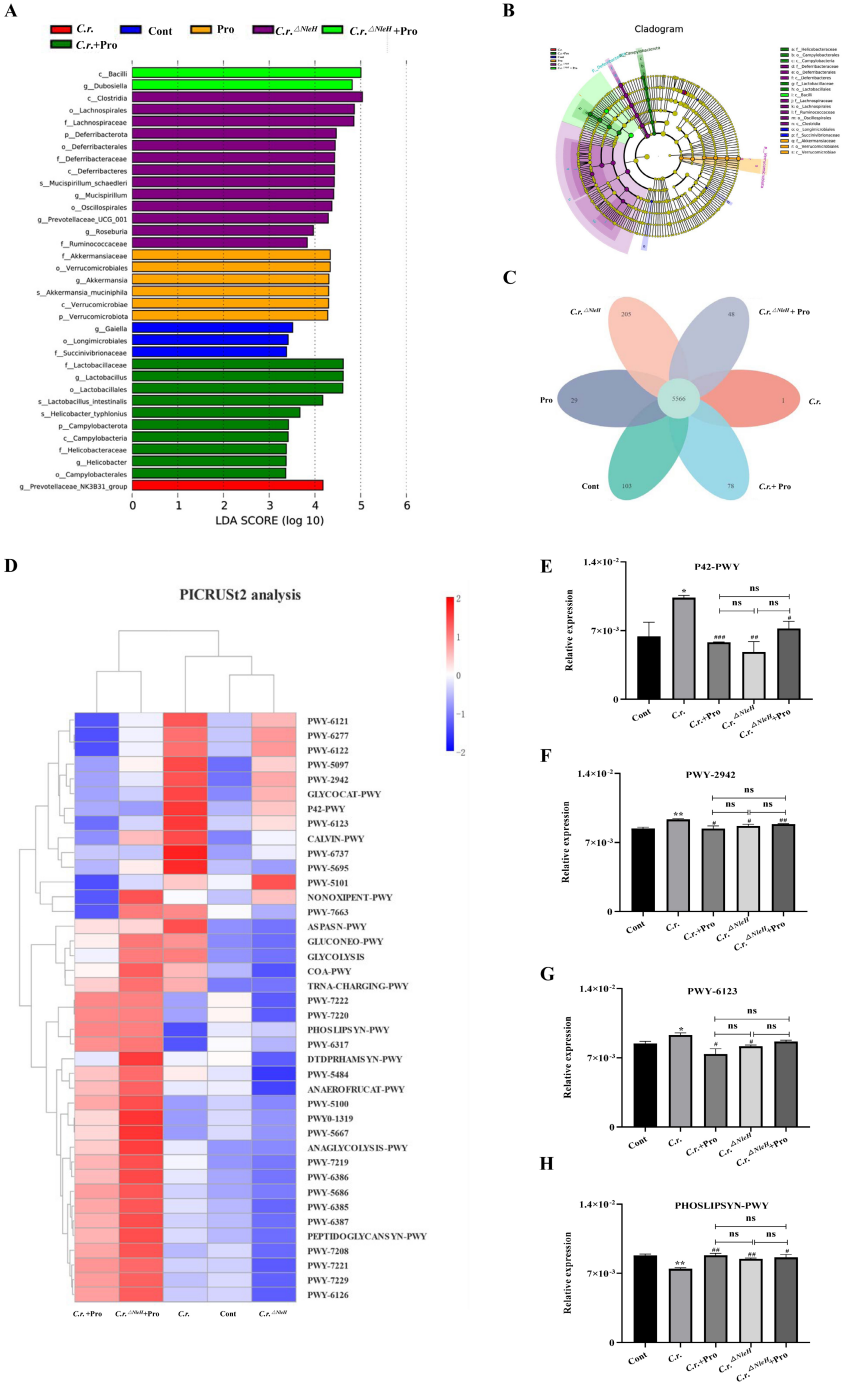
Metabolic function analysis of intestinal microbiota. (A, B) The contributor species for the differential metabolic pathways (Lefse analysis). (C) Wayne plots of pathway levels in different sample groups. (D) Clustering heat map of metabolic function of intestinal microbiota. The functional relative expression of intestinal microbiota involved in the tricarboxylic acid cycle pathway (E), L‐lysine biosynthesis pathway (F), de novo purine biosynthesis pathway (G) and phospholipid biosynthesis pathway (H) in each group. *n* = 4, *p < 0.05, **p < 0.01 versus Cont; # *p* < 0.05, ## *p* < 0.01 versus *C.r*.

We analysed the microbiome and metabolic function of mouse faecal samples. The gut microbiota of all groups was involved in 6030 metabolic pathways (Figure 5A–C). Functional abundance cluster heat maps showed enhanced tricarboxylic acid cycle, L‐lysine biosynthesis, and de novo purine biosynthesis pathways in *C.r*. group mice. However, in the *C.r*. + Pro, *C.r*.^*△NleH*^, and *C.r*.^*△NheH*^ + Pro groups, these functions were diminished (Figure 5D–H). In contrast, the microbiota of mice in the *C.r*. group showed a reduced capacity for phospholipid biosynthesis, while this function was upregulated in the *C.r*. + Pro, *C.r*.^*△NleH*^, and *C.r*.^*△NheH*^ + Pro groups (Figure 5H). The major contributing species of differential metabolic pathways are *Campylobacterota*, *Deferribacterota* and *Verrucomicrobiota* (Figure 5A).

## 4 Discussion

Metabolites derived from the gut microbiota mediate interactions with the host and help regulate host health and disease. SCFAs is a major product fermented from dietary fibre by gut microbiota and plays an important role in regulating mucosal barrier integrity, regulating mucosal immune system activity and maintaining epithelial energy metabolism [17, 18]. Propionate is a major SCFAs with unique characteristics that affect gut health, although it has been less studied compared to other microbial metabolites such as butyrate and acetate [19]. Research has shown that propionate mediated immune and metabolic signal transduction is associated with various chronic inflammatory diseases and metabolic disorders. It is worth noting that increasing the production of propionate in the colon through dietary intervention is a potential strategy for maintaining host immunity and metabolic homeostasis [20, 21, 22]. Diversified dietary interventions that alter dietary fibre have been shown effects on gut microbiota and improve the health status of participants. However, the exact mechanisms behind these observations remain elusive, especially at the molecular pathway level of bacteria. The important question to be addressed in this study is whether propionate affects bacterial colonisation and infection, thereby improving intestinal health.

The interaction between bacteria and intestinal epithelial cells is an important prerequisite for its pathogenesis. Given the above considerations, we first investigated the effect of propionate on the growth and adhesion of *C. rodentium* . NleH/NleH1 mutant was co‐cultured with propionate in vitro and detect the growth status of the strains. We found that NleH/NleH1 knockout did not affect strains growth, while propionate significantly inhibited the growth of strains. Animal experiments have shown that propionate can effectively intervene in intestinal problems caused by *C. rodentium* infection. Our results showed that propionate can significantly alleviate the degree of weight loss in infected mice and reduce the intestinal bacterial excretion, which was related to the expression of NleH in *C. rodentium* . Meanwhile, the NleH mutant strains showed decreased infectivity and did not respond to propionate intervention. The above results preliminarily confirmed that the anti‐inflammatory properties of propionate were related to NleH expression in *C. rodentium* . By comparing the pathological changes of colonic inflammation in mice from different treatment groups, it was also found that the effect of propionate in reducing colonic mucosal damage was related to NleH. The colonic damage induced by NleH mutant was weaker than that of wild‐type strains, and propionate intervention could not effectively alleviate the inflammatory damage induced by NleH mutant.

The integrity of the intestinal epithelial barrier function is crucial for host resistance to pathogen invasion. ZO‐1 and occludin are tight junction proteins in intestinal epithelium, involved in maintaining intestinal barrier function [23, 24]. By detecting the levels of tight junction proteins in colon of infected mice, our results showed that *C. rodentium* infection induced a decrease in ZO‐1 and occludin levels, while the expression of these tight junction proteins increased in the NleH mutant infection and propionate intervention groups. However, it is worth noting that propionate does not seem to have a significant effect on the expression of Muc2 (results were not shown), which may be related to the complex role of Muc2 in intestinal inflammation [25].

Given that NleH is a key virulence factor associated with *C. rodentium* colonisation, its low expression level can reduce bacterial colonisation in the gut [10]. Our study first investigated the effect of propionate regulating NleH on bacterial adhesion in vitro, and results showed that NleH/NleH1 mutants had lower adhesion ability than wild‐type strains and was not responsive to propionate. By measuring the amount of bacterial invasion in different organs of mice in each group, the results also showed that propionate indeed weakened the invasion potential of *C. rodentium* in vivo, and this effect was achieved through its regulation of NleH expression.

Gut microbiota is the central regulator of host metabolism. The composition and function of the gut microbiota are dynamic and influenced by multiple factors. Metabolites derived from microbial communities can metabolise substrates and signalling molecules in the host gut, which can have an impact on host metabolism and health [26]. In this study, through the metabolic function analysis of gut microbiota, we found that among the 6030 metabolic pathways involved in the microbiota, *C. rodentium* infection induced increased metabolic activity of the tricarboxylic acid cycle, L‐lysine synthesis and de novo purine biosynthesis, while decreased metabolic activity of the phospholipid biosynthesis. On the contrary, the changes of the above metabolic pathways were reversed in the propionate intervention and NleH mutant infection groups. Relevant studies have confirmed that the above pathways play a key role in maintaining intestinal health and resisting pathogenic infections [27]. In conclusion, the regulation of propionate on intestinal microbiota metabolism become a potential key mechanism for exploring its alleviation of *C. rodentium* infection.

## 5 Conclusion

**FIGURE 6:**
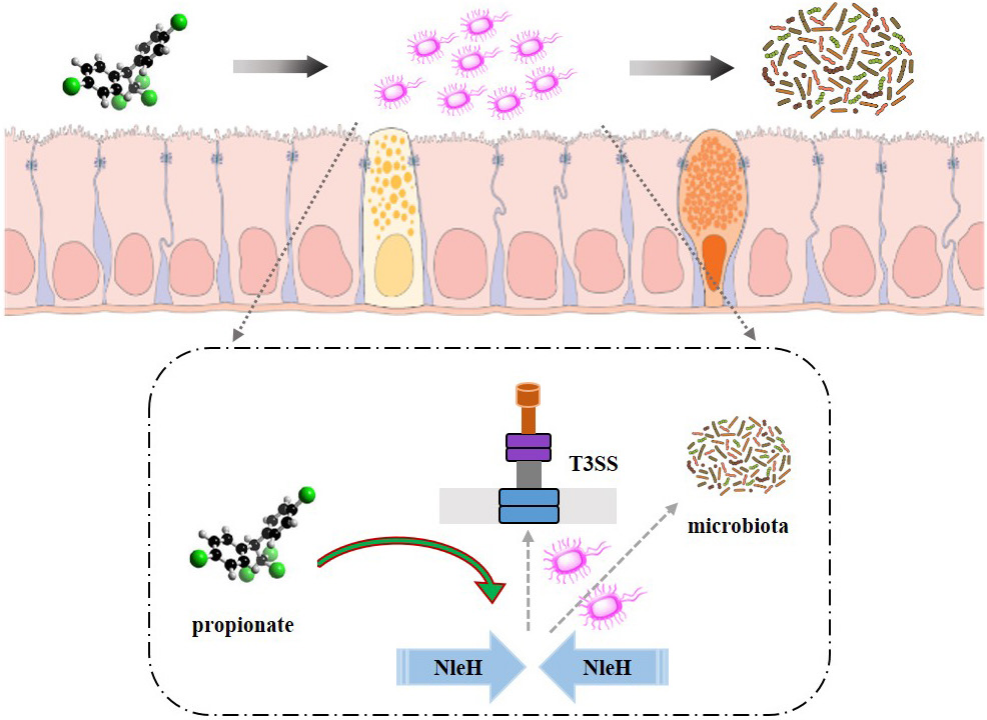
Mechanism diagram of propionate affecting *C. rodentium* infection by regulating NleH. T3SS, type III secretion system.

In conclusion, propionate intervenes in intestinal issues caused by *C.r*. infection by down‐regulating NleH expression, which through multiple pathways including enhancement of intestinal epithelial barrier function, inhibition of bacterial adhesion and modulation of gut microbiota metabolic functions (Figure 6). These findings provide robust support for the application of propionate in the prevention and treatment of intestinal diseases, and establish a foundation for further exploring its underlying mechanisms of action.

## Author Contributions

**Yingying Li:** data curation (equal), investigation (equal), methodology (equal), supervision (equal), writing – original draft (equal), writing – review and editing (equal). **Wenjie Mei:** methodology (supporting). **Qinhan Zhang:** methodology (supporting). **Li Li:** conceptualization (equal), funding acquisition (equal), writing – review and editing (equal). **Qiaorong Ji:** conceptualization (lead), data curation (equal), funding acquisition (equal), methodology (lead), project administration (lead), supervision (equal), writing – original draft (lead), writing – review and editing (lead).

## Conflicts of Interest

The authors declare no conflicts of interest.

## Data Availability

The data that support the findings of this study are available from the corresponding author upon reasonable request.

## References

[1] Antimicrobial Resistance Collaborators , “Global Burden of Bacterial Antimicrobial Resistance in 2019: A Systematic Analysis,” Lancet 399, no. 10325 (2022): 629–655, 10.1016/S0140-6736(21)02724-0. 35065702 PMC8841637

[2] C. Zhang , X. Fu , Y. Liu , H. Zhao , and G. Wang , “Burden of Infectious Diseases and Bacterial Antimicrobial Resistance in China: A Systematic Analysis for the Global Burden of Disease Study 2019,” Lancet Regional Health. Western Pacific 43 (2023): 100972, 10.1016/j.lanwpc.2023.100972. 38076321 PMC10700598

[3] N. Bouladoux , O. J. Harrison , and Y. Belkaid , “The Mouse Model of Infection With *Citrobacter rodentium* ,” Current Protocols in Immunology 119 (2017): 19.15.1–19.15.25, 10.1002/cpim.34. PMC5667658 29091261

[4] T. Mao , C. W. Su , Q. Ji , et al., “Hyaluronan‐Induced Alterations of the Gut Microbiome Protects Mice Against *Citrobacter rodentium* Infection and Intestinal Inflammation,” Gut Microbes 13, no. 1 (2021): 1972757, 10.1080/19490976.2021.1972757. 34592891 PMC8489935

[5] J. Zhang , Z. Guo , Z. Xue , et al., “A Phylo‐Functional Core of Gut Microbiota in Healthy Young Chinese Cohorts Across Lifestyles, Geography and Ethnicities,” ISME Journal 9, no. 9 (2015): 1979–1990, 10.1038/ismej.2015.11. 25647347 PMC4542028

[6] J. Ma , X. Piao , S. Mahfuz , S. Long , and J. Wang , “The Interaction Among Gut Microbes, the Intestinal Barrier and Short Chain Fatty Acids,” Animal Nutrition 9 (2021): 159–174, 10.1016/j.aninu.2021.09.012. 35573092 PMC9079705

[7] C. J. Hong , S. Y. Chen , Y. H. Hsu , and G. C. Yen , “Protective Effect of Fermented Okara on the Regulation of Inflammation, the Gut Microbiota, and SCFAs Production in Rats With TNBS‐Induced Colitis,” Food Research International 157 (2022): 111390, 10.1016/j.foodres.2022.111390. 35761646

[8] J. G. Lee , J. Lee , A. R. Lee , et al., “Impact of Short‐Chain Fatty Acid Supplementation on Gut Inflammation and Microbiota Composition in a Murine Colitis Model,” Journal of Nutritional Biochemistry 101 (2022): 108926, 10.1016/j.jnutbio.2021.108926. 34848335

[9] G. Wang , L. A. Feuerbacher , and P. R. Hardwidge , “Influence of Intestinal Microbiota Transplantation and NleH Expression on *Citrobacter rodentium* Colonization of Mice,” Pathogens 7, no. 2 (2018): 35, 10.3390/pathogens7020035. 29601470 PMC6027419

[10] V. A. García‐Angulo , W. Deng , N. A. Thomas , B. B. Finlay , and J. L. Puente , “Regulation of Expression and Secretion of NleH, a New Non‐Locus of Enterocyte Effacement‐Encoded Effector in *Citrobacter rodentium* ,” Journal of Bacteriology 190, no. 7 (2008): 2388–2399, 10.1128/JB.01602-07. 18223087 PMC2293213

[11] T. H. Pham , X. Gao , K. Tsai , R. Olsen , F. Wan , and P. R. Hardwidge , “Functional Differences and Interactions Between the *Escherichia coli* Type III Secretion System Effectors NleH1 and NleH2,” Infection and Immunity 80, no. 6 (2012): 2133–2140, 10.1128/IAI.06358-11. 22451523 PMC3370600

[12] C. S. Byrne , E. S. Chambers , H. Alhabeeb , et al., “Increased Colonic Propionate Reduces Anticipatory Reward Responses in the Human Striatum to High‐Energy Foods,” American Journal of Clinical Nutrition 104, no. 1 (2016): 5–14, 10.3945/ajcn.115.126706. 27169834 PMC4919527

[13] C. Hemrajani , O. Marches , S. Wiles , et al., “Role of NleH, a Type III Secreted Effector From Attaching and Effacing Pathogens, in Colonization of the Bovine, Ovine, and Murine Gut,” Infection and Immunity 76, no. 11 (2008): 4804–4813, 10.1128/IAI.00742-08. 18725419 PMC2573362

[14] S. V. Royan , R. M. Jones , A. Koutsouris , et al., “Enteropathogenic *E. coli* Non‐LEE Encoded Effectors NleH1 and NleH2 Attenuate NF‐κB Activation,” Molecular Microbiology 78, no. 5 (2010): 1232–1245, 10.1111/j.1365-2958.2010.07400.x. 21091507 PMC3325542

[15] S. E. Kralicek , M. Nguyen , K. J. Rhee , R. Tapia , and G. Hecht , “EPEC NleH1 Is Significantly More Effective in Reversing Colitis and Reducing Mortality Than NleH2 via Differential Effects on Host Signaling Pathways,” Laboratory Investigation; a Journal of Technical Methods and Pathology 98, no. 4 (2018): 477–488, 10.1038/s41374-017-0016-1. 29396422 PMC5920738

[16] Q. Ji , Y. Zhang , Y. Zhou , et al., “Effects of Hypoxic Exposure on Immune Responses of Intestinal Mucosa to Citrobacter Colitis in Mice,” Biomedicine & Pharmacotherapy 129 (2020): 110477, 10.1016/j.biopha.2020.110477. 32768962

[17] X. Wang , Z. Cai , Q. Wang , et al., “Bacteroides Methylmalonyl‐CoA Mutase Produces Propionate That Promotes Intestinal Goblet Cell Differentiation and Homeostasis,” Cell Host & Microbe 32, no. 1 (2024): 63–78, 10.1016/j.chom.2023.11.005. 38056459

[18] A. Koh , F. De Vadder , P. Kovatcheva‐Datchary , and F. Bäckhed , “From Dietary Fiber to Host Physiology: Short‐Chain Fatty Acids as Key Bacterial Metabolites,” Cell 165, no. 6 (2016): 1332–1345, 10.1016/j.cell.2016.05.041. 27259147

[19] E. Hosseini , C. Grootaert , W. Verstraete , and T. Van de Wiele , “Propionate as a Health‐Promoting Microbial Metabolite in the Human Gut,” Nutrition Reviews 69, no. 5 (2011): 245–258, 10.1111/j.1753-4887.2011.00388.x. 21521227

[20] G. den Besten , A. Bleeker , A. Gerding , et al., “Short‐Chain Fatty Acids Protect Against High‐Fat Diet‐Induced Obesity via a PPARγ‐Dependent Switch From Lipogenesis to Fat Oxidation,” Diabetes 64, no. 7 (2015): 2398–2408, 10.2337/db14-1213. 25695945

[21] N. M. McKeown , G. C. Fahey, Jr. , J. Slavin , and J. W. van der Kamp , “Fibre Intake for Optimal Health: How Can Healthcare Professionals Support People to Reach Dietary Recommendations?,” BMJ 378 (2022): e054370, 10.1136/bmj-2020-054370. 35858693 PMC9298262

[22] A. Duscha , B. Gisevius , S. Hirschberg , et al., “Propionic Acid Shapes the Multiple Sclerosis Disease Course by an Immunomodulatory Mechanism,” Cell 180, no. 6 (2020): 1067–1080, 10.1016/j.cell.2020.02.035. 32160527

[23] R. Al‐Sadi , K. Khatib , S. Guo , D. Ye , M. Youssef , and T. Ma , “Occludin Regulates Macromolecule Flux Across the Intestinal Epithelial Tight Junction Barrier,” American Journal of Physiology. Gastrointestinal and Liver Physiology 300, no. 6 (2011): G1054–G1064, 10.1152/ajpgi.00055.2011. 21415414 PMC3119114

[24] W. T. Kuo , M. A. Odenwald , J. R. Turner , and L. Zuo , “Tight Junction Proteins Occludin and ZO‐1 as Regulators of Epithelial Proliferation and Survival,” Annals of the New York Academy of Sciences 1514, no. 1 (2022): 21–33, 10.1111/nyas.14798. 35580994 PMC9427709

[25] Y. Liu , X. Yu , J. Zhao , H. Zhang , Q. Zhai , and W. Chen , “The Role of MUC2 Mucin in Intestinal Homeostasis and the Impact of Dietary Components on MUC2 Expression,” International Journal of Biological Macromolecules 164 (2020): 884–891, 10.1016/j.ijbiomac.2020.07.191. 32707285

[26] M. Schoeler and R. Caesar , “Dietary Lipids, Gut Microbiota and Lipid Metabolism,” Reviews in Endocrine & Metabolic Disorders 20, no. 4 (2019): 461–472, 10.1007/s11154-019-09512-0. 31707624 PMC6938793

[27] W. M. de Vos , H. Tilg , M. Van Hul , and P. D. Cani , “Gut Microbiome and Health: Mechanistic Insights,” Gut 71, no. 5 (2022): 1020–1032, 10.1136/gutjnl-2021-326789. 35105664 PMC8995832

